# Ligand exchange method for determination of mole ratios of relatively weak metal complexes: a comparative study

**DOI:** 10.1186/s13065-018-0512-4

**Published:** 2018-12-20

**Authors:** Mokhtar Mabrouk, Sherin F. Hammad, Mohamed A. Abdelaziz, Fotouh R. Mansour

**Affiliations:** 1 Department of Pharmaceutical Analytical Chemistry, Faculty of Pharmacy, Tanta University, Tanta, 31111 Egypt; 2 Pharmaceutical Services Center, Faculty of Pharmacy, Tanta University, Tanta, 31111 Egypt; 3 Department of Pharmaceutical Analytical Chemistry, Faculty of Pharmacy, Kafrelsheikh University, Kafrelsheikh, 33511 Egypt

**Keywords:** Ligand exchange method, Mole ratio method, Job’s method, Bisphosphonates, Relatively weak complexes

## Abstract

Ligand exchange method is introduced as an alternative to Job’s and mole ratio methods for studying the stoichiometry of relatively weak metal complexes in solutions. The method involves adding varying amounts of a ligand (L) to an excess constant amount of a colored complex (MX) with appropriate stability and molar absorptivity. The absorbance of each solution is measured at the λ_max_ of the initial complex, MX, and plotted against the concentration of the studied ligand, L. If the newly formed complex ML does not absorb at the λ_max_ of the initial complex, then attenuation of the absorbance of the initial complex on adding varying quantities of the investigational ligand gives an inverse calibration line that intersects with the calibration curve of initial complex at a given point. If a line parallel to the ordinate is drawn from this point to the x-axis, the ratio of the two parts of the x-axis to the left and to the right (α/β) gives the metal to ligand molar ratio in the complex formed, ML. The new method has been applied to the study of the composition of iron (III) complexes with three bisphosphonate drugs: alendronate, etidronate, and ibandronate. The mole ratio was found to be 1:1 with the three investigated bisphosphonates and results were further confirmed by Job’s and mole ratio methods. The ligand exchange method is simpler, quicker, easier to perform and more accurate than Job’s and mole ratio methods for studying weak and relatively weak complexes.

**Electronic supplementary material:**

The online version of this article (10.1186/s13065-018-0512-4) contains supplementary material, which is available to authorized users.

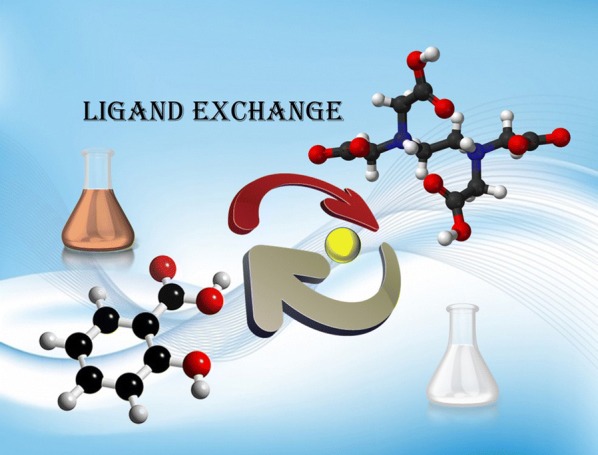

## Introduction

The mole ratio is the proportion of number of moles of any two chemical entities involved in a compound or a chemical reaction. Studying the mole ratio is important to calculate the reaction yield, determine the stoichiometry and monitor the reaction kinetics. Several spectrophotometric methods were developed for the determination of the molar ratio of metal complexes. The first method goes back to the contributions of Ostromisslensky [1] and Job [2], and was widely known as Job’s method of continuous variations. In this method, a series of solutions are prepared by mixing varying proportions of the metal and ligand, keeping the sum of the total molar concentrations constant. The absorbance of each solution is then plotted against the mole fraction of either the ligand or metal. The position of the maximum in the resulting curve, or minimum in some cases [3], gives the mole fraction. The simplicity of the method made it widely applied for the study of various metals and association complexes [4–9], in spite of its limitations. For instance, strong complexes give triangular plots from which the position of the maximum is easily determined, while the plots of weak complexes are highly curved leading to unreliable results. Normalized absorbance plots (A/A_max_ vs. mole fraction) gave sharper plots at the maxima and allowed for better location of the mole ratio [10], but for weak complexes, these normalized Job plots were still highly curved.

Besides the method of continuous variations, the mole ratio method has been used frequently since its introduction by Yoe and Jones [11]. In this method, a series of solutions are prepared by varying the amount of ligand in each solution while the amount of metal is kept constant. If a stable complex is formed, a plot of absorbance versus mole ratio of ligand to metal (L/M) gives a straight line that rises until it reaches the point corresponding to the mole ratio (L/M), then it breaks to a differently sloped line. For moderately stable complex, the mole ratio corresponds to the point of intersection of the tangents of straight-line portions of the plot. However, if a weak complex is formed, a very curved plot is obtained, making the identification of the molar ratio of these complexes uncertain. As a result, several chemical [12] and mathematical modifications [13–15] have been made to the basic mole ratio method so that it can reliably be applied to study the composition of weak complexes. However, these modifications make the method relatively more complicated and are only applicable when the ligand has significant absorbance which is not always the case.

**Fig. 1:**
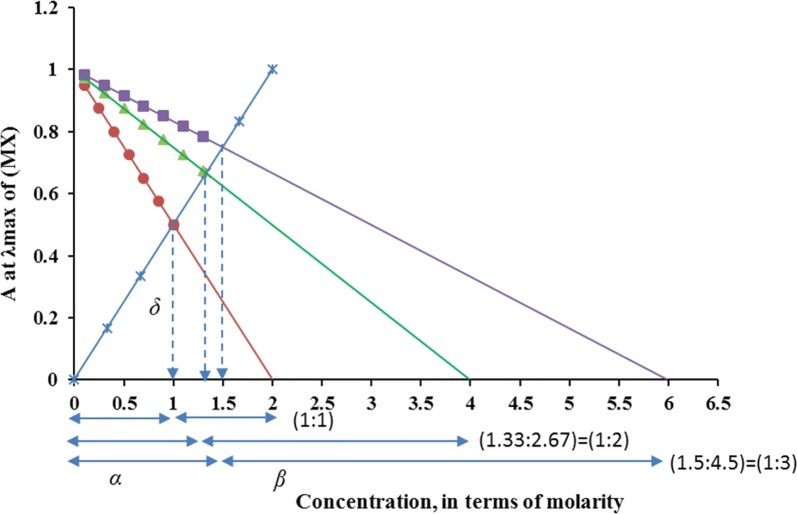
Illustrative plots of the ligand exchange method using MX as an initial complex (*) for studying the mole ratios of complexes: ML (●), ML_2_ (▲), and ML_3_ (■)

A recent method based on ligand exchange has been introduced by Mansour and Danielson [16]. The method involves adding varying amounts of the ligand (L), whose combining ratio with metal (M) is being studied, to an excess constant amount of a colored complex (MX) with appropriate stability and molar absorptivity. The absorbance of each solution is measured at the λ_max_ of the initial complex, MX, and plotted against the concentration of the studied ligand, L. If the newly formed complex, ML, does not absorb at the λ_max_ of the initial complex, then attenuation of the absorbance of the initial complex on adding varying quantities of the investigational ligand gives an inverse calibration line that intersects with the calibration curve of initial complex at a given point (Fig. 1). If a line parallel to the ordinate is drawn from this point to the x-axis, the ratio of the two parts of the x-axis to the left and to the right (α/β) gives the metal to ligand molar ratio in the complex formed. A video that explains the principle of Mansour-Danielson's method is shown in Additional file 1.

**Fig. 2:**
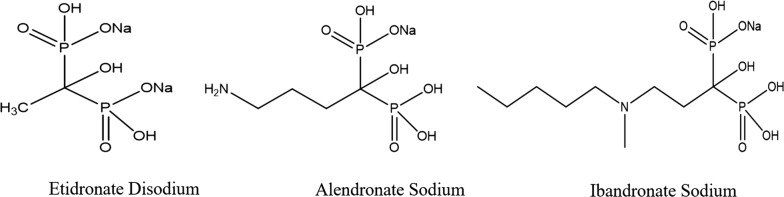
Molecular structures of studied bisphosphonate drugs. All compounds are presented in anhydrous forms

In our previous work, the ligand exchange method has been applied for determination of mole ratios other than 1:1 [16]. In this work, we present the mathematical proof of the ligand exchange method for the first time and apply it for determination of relatively weak complexes of selected bisphosphantes (Fig. 2) with ferric ion [9]. The ferric complexes of bisphosphonates are used for the spectrophotometric determination of bisphosphonates in pharmaceutical tablets [9]. Determination of the mole ratios of these complexes is important to adjust the amount added of the ferric salt in the experimental part. The ligand exchange method was also compared with Job’s and mole ratio methods; its advantages over these commonly employed methods are discussed.

## Theory of Mansour–Danielson’s method of ligand exchange

Suppose that MX and ML are two complexes of a metal M with two ligands, X and L, where MX is a colored complex, ML is a colorless complex and MX is less stable than ML. For a certain concentration of the complex MX, the absorbance depends on the molar absorptivity of MX (*ε*_*MX*_) and the concentration (*C*_*MX*_) according to the equation:

1 $$A = \varepsilon_{MX} \cdot C_{MX }$$

If a certain amount of ligand L was added to the previous MX solution, a displacement reaction will take place and the absorbance will decrease as shown in Fig. 1. The decrease in the absorbance depends on the concentration of the ligand L (*C*_*L*_) and the mole ratio of the ML complex (*n*) according to the equation:

2 $$A = \varepsilon_{MX} \cdot \,(C_{MX} - nC_{L} )$$

From Eq. 2, we get:

3 $$A = \varepsilon_{MX} \cdot C_{MX} - n \, \varepsilon_{MX} \cdot C_{L }$$

Equation 3 is a straight line equation (y = a ± bx) with an intercept equals *ε*_*MX*_*·C*_*MX*_ and a slope equals −*n*·*ε*_*MX*_. If *A* was plotted against *C*_*L*_, a straight line with a negative slope will be obtained as shown in Fig. 1. The mole ratio can be determined graphically from the overlay of the two calibration curves as follows:

A straight line parallel to the y-axis is drawn from the intersection point of the calibration curves to divide the x-axis into two parts: *α* and *β*. The length of both parts (*α* and *β*) can be calculated from the length of the parallel line (*δ*) and the slopes of the calibration curves where:

4 $$\alpha = \frac{\delta }{Slope \, of \, Eq1} = \frac{\delta }{{\varepsilon_{\text{MX}} }}$$

while,

5 $$\beta = \frac{\delta }{Slope \, of \, Eq2} = \frac{\delta }{{n\varepsilon_{\text{MX}} }}$$

From Eqs. 4 and 5, we get:

6 $$\frac{\alpha }{\beta } = n$$

## Experimental

### Instrumentation

*Jenway 3510 (Jenway, UK) and Biochrom libra S80 (Biochrom, Cambridge, UK)* were employed in all pH and absorbance measurements, respectively.

### Materials

Alendronate sodium trihydrate, etidronate disodium, and ibandronate sodium monohydrate of pharmaceutical grade were kindly provided by Sigma Pharmaceutical Industries (Quesna, Menofyia, Egypt). All other chemicals and solvents used were of analytical ACS grade, purchased from Fisher Scientific (Fair Lawn, NJ, USA) and Sigma-Aldrich (St. Louis, MO, USA).

### Standard solutions

Fe(III)-salicylate solution was prepared at 10 mM in water/methanol (50:50, pH 3.2) and was proved to be stable for months when kept refrigerated. Fe(III) chloride stock solution (for the mole ratio and Job’s methods) was prepared at 10 mM in 2 M HClO_4_. Etidronate disodium stock solution was prepared at 10 mM in two different diluents: 2 M HClO_4_ for both the mole ratio and Job’s methods and water/methanol (50:50, pH 3.2) for the ligand exchange method. Similarly, stock solutions of alendronate sodium and ibandronate sodium were prepared.

### Procedures

#### Ferric salicylate complex calibration curve

A series of standard solutions of ferric salicylate in the range of 0.1–0.6 mM were prepared by accurately transferring appropriate aliquots of ferric salicylate stock solution (10 mM) into a series of 10 mL calibrated volumetric flasks, then completed to the mark with water/methanol (50:50, pH 3.2) (Ionic strength was adjusted with 0.5 M NaCl). Absorbance at 535 nm was measured and plotted against ferric salicylate concentration.

#### Ligand exchange method

Aliquots in the range 0.2–1.8 µmol of etidronate disodium were accurately transferred into a series of 10 mL volumetric flasks containing 3 µmol ferric salicylate, then completed to the mark with water/methanol (50:50, pH 3.2) (Ionic strength was adjusted with 0.5 M NaCl). Absorbance at 535 nm was measured and plotted against concentration. A similar procedure was applied to determine the mole ratio of Fe(III)-alendronate and Fe(III)-ibandronate.

#### Job’s method

Standard nine mixtures of ferric chloride (in 2 M HClO_4_) and etidronate (in 2 M HClO_4_) were prepared by adding aliquots of Fe(III) equivalent to 1 − 9 µmol into a series of 10 mL volumetric flasks containing aliquots of etidronate equivalent to 9 − 1 µmol so that each flask contains a total number of 10 µmol. Each flask is completed to the mark using HClO_4_ (2 M). Job’s graph is obtained by plotting absorbance at 300 nm against the mole fraction of Fe(III) ion. The same procedure was repeated with ibandronate and alendronate.

#### Mole ratio method

Standard mixtures of ferric chloride (in 2 M HClO_4_) and etidronate (in 2 M HClO_4_) were prepared by adding aliquots of Fe(III) equivalent to 0.4–30 µmol into a series of 10 mL volumetric flasks containing 5 µmol of etidronate. Each flask is completed to the mark using HClO_4_ (2 M). The mole ratio graph is obtained by plotting absorbance at 300 nm against the mole ratio (Fe(III)/etidronate). The same procedure was applied to study the stoichiometry of Fe(III)-ibandronate and Fe(III)-alendronate.

## Results and discussion

### Absorption spectra

**Fig. 3:**
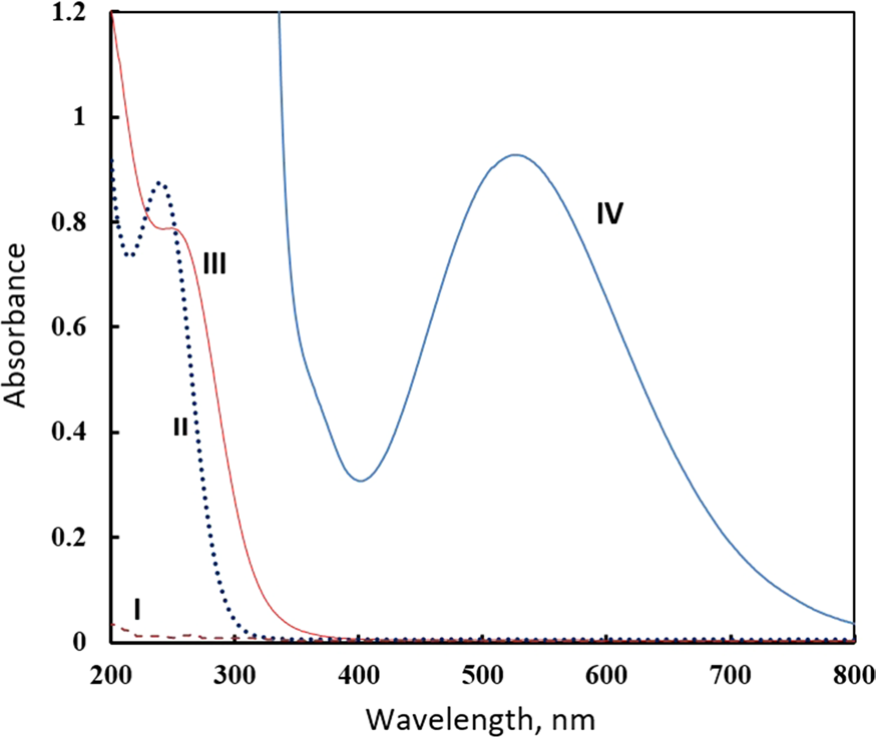
Absorption spectra of (I) etidronate (1 × 10^−3^ M), (II) FeCl_3_ (2 × 10^−4^ *M*), and (III) FeCl_3_ (2 × 10^−4^ M) + etidronate (4 × 10^−4^ M) all in 2 M perchloric in addition to (IV) the absorption Spectrum of Fe(III)-salicylate in water/methanol (50:50, pH 3.2)

The absorption spectra of reacting species, Fe(III) ions and etidronate, together with the absorption spectrum of their complex have been recorded in 2 M perchloric acid in the wavelength range from 200 to 400 nm (Fig. 3). Spectra of iron(III) perchlorate and iron(III)-etidronate complex show an absorption maximum at 239 and 252 nm, respectively. On the other hand, etidronate and the other studied bisphosphonates do not show significant absorbance in the spectral region indicated above [17]. For Job’s and mole ratio methods, all absorbance measurements were performed at 300 nm where the absorbance difference between the complex and Fe(III) ions approaches maximum, and the absorption of metal ions is low. For the ligand exchange method, all spectrophotometric measurements were conducted at 535 nm, the wavelength that corresponds to the absorption maximum of iron(III)-salicylate at the conditions employed.

### Ligand exchange method using Fe(III)-salicylate

According to a previously published work that studied the effect of pH and ionic strength on the absorbance of Fe(III)-salicylate complex [18], the absorbance of the complex was found constant over a pH range of (2.5–3.5). After trying several solvents, a 50% methanol at pH 3.2 was chosen owing to the high Fe(III)-salicylate absorbance and reasonable plateau that ensures the robustness of the method against small changes in pH. A solution of 0.5 M NaCl was used to adjust the ionic strength and keep it constant over all the following procedures.

**Fig. 4:**
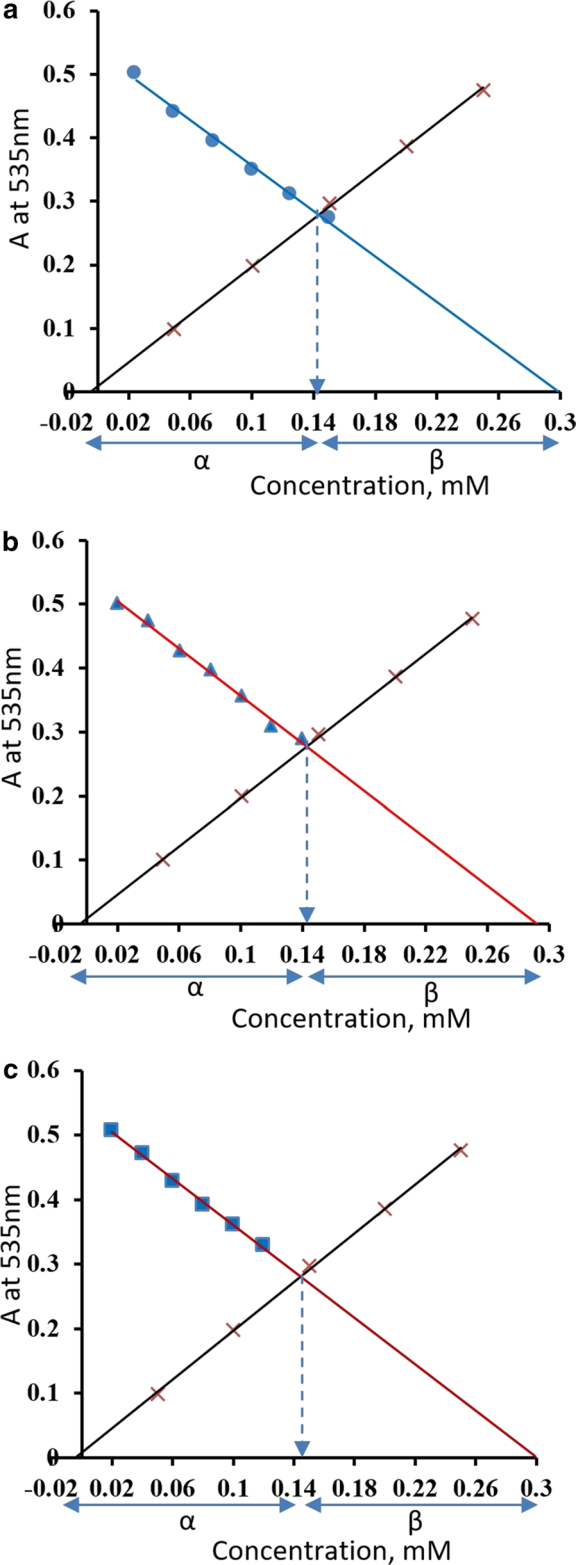
An overlay of Fe(III)-salicylate calibration curve (×) with inverse calibration curves of **a** ibandronate (●), **b** alendronate (▲), and **c** etidronate (■)

An overlay of the direct and inverse calibration curves of ferric salicylate and bisphosphonate, respectively, is used to determine the combining metal to ligand ratio (Fig. 4). The quotient of *α*/*β* is equal to the stoichiometric ratio of metal to bisphosphonate ligand and was found to be 1:1 with the three investigated bisphosphonates. Calibration curves of the three studied bisphosphonates were linear in the range (0.02–0.18) mM with correlation coefficients (r) equal − 0.999, − 0.997 and − 0.996 with etidronate, alendronate, and ibandronate, respectively.

### Comparison to other mole ratio methods

**Fig. 5:**
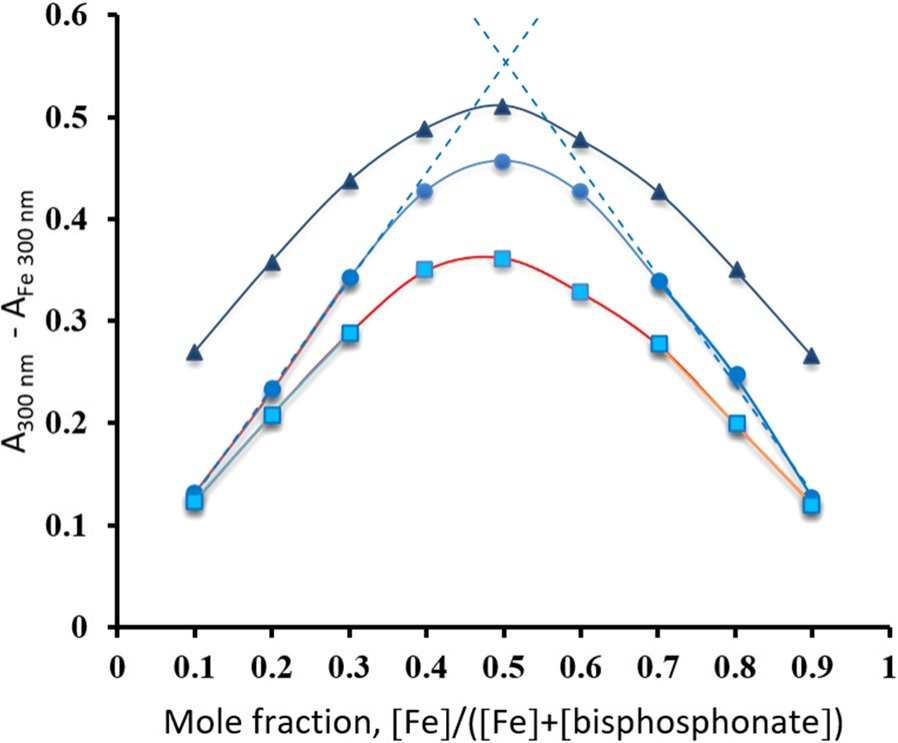
Job plots of Fe(III) complexes with etidronate (■), alendronate (▲), and ibandronate (●) ([Fe(III)] + [bisphosphonate]) = 1 mM

**Fig. 6:**
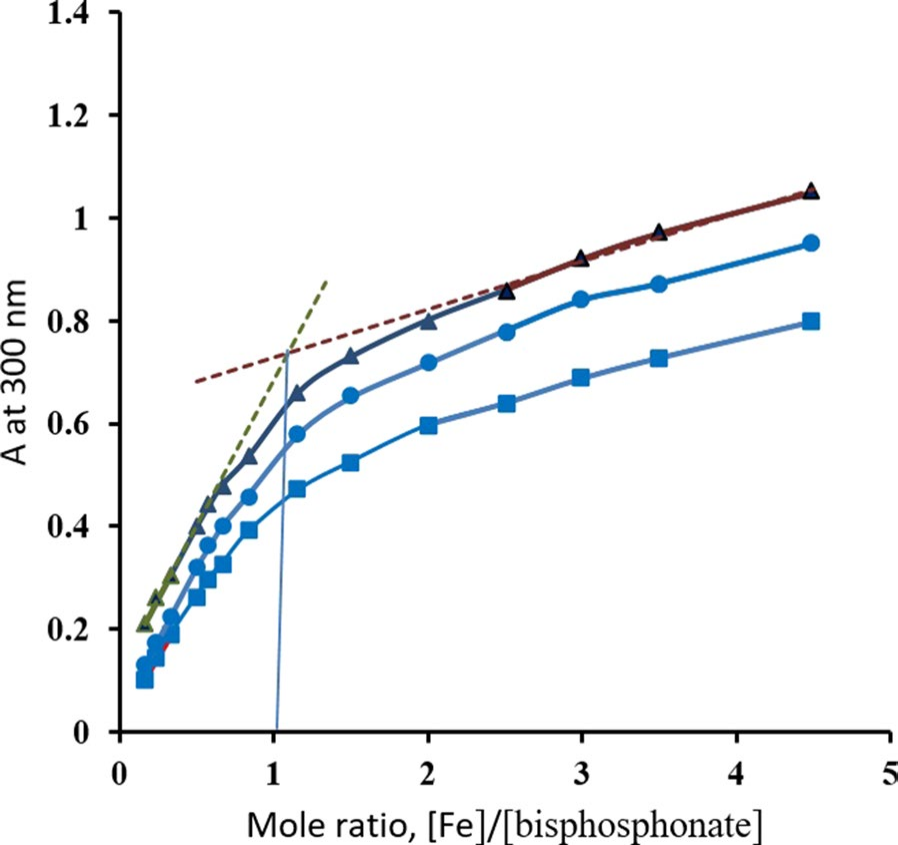
Molar ratio method: plots of Fe(III) complex with etidronate (■), alendronate (▲) and ibandronate (●) ([bisphosphonate] = 0.5 mM)

The 1:1 ratio determined for the Fe(III) complex with alendronate is congruent with the work of Kuljanin and his colleagues [9] that is based on Job’s and mole ratio methods. On the other hand, results of ibandronate and etidronate complexes with Fe(III) have been confirmed by performing Job’s and mole ratio methods. The Job’s plots (Fig. 5) showed a peak at a mole fraction of 0.5, whereas the tangents of straight-line portions of the mole ratio curves intersect at a value of 1 (Fig. 6). Therefore, results of both methods provide a further confirmation of the 1:1 ratio determined by the ligand exchange method.

Compared to the Job and mole ratio methods, the ligand exchange method offers several advantages: (i) it enables the study of the composition of colorless metal complexes using a colorimetric technique and the green LED lamp that is commercially available in most colorimeters (ii) it requires fewer steps than Job’s and the mole ratio methods because fewer number of points can be adequate to plot a straight line and several ligands can be studied against a single calibration curve of the initial complex, (iii) the ligand exchange method is more accurate and more precise than Job’s and the mole ratio methods for determination of weak and relatively weak complexes; determining the mole ratio using these methods in this case is subjective due to the curved lines. As shown in Additional file 2: Fig. S1, different tangents can be drawn for the same group of points, which may lead to false conclusions while in the ligand exchange method, there is no need to draw tangents which obviates bias and decreases the risk of error. (iv) The ligand exchange method could be used for metals other than ferric, such as Cu(II), and for determination of mole ratios other than 1:1 [16] which indicates the generality of the method and (v) neither Job’s nor the mole ratio methods can be used unless one of the studied reactants or the formed complex are absorbing. In this case, the ligand exchange will be the method of choice.

## Conclusion

The ligand exchange method can reliably be used as an alternative to Job’s and mole ratio methods for the determination of formula of complexes with the aid of a simple colorimeter, and could be superior in determining the composition of weak and relatively weak complexes. The method has successfully been applied to the study of the composition of ferric ion complexes with the non-chromophoric bisphosphonates: alendronate, etidronate and ibandronate. The ligand exchange method gives straight lines from which the exact mole ratio can be determined. The method does not require tangent drawing which can be subjective and may lead to inaccurate conclusions especially when weak complexes are studied. The ligand exchange method could also be preferable for determining the composition of high ratio complexes and that will be the focus of our future research.

## Additional files

**Additional file 1:** A video that explains the principle of Mansour-Danielson's method.

**Additional file 2: Fig. S1.** Molar ratio’s plots for Fe(III) complex with ibandronate showing different conclusions for the same results depending on the drawn tangents.

## References

[1] Ostromisslensky I (1911). Über eine neue, auf dem Massenwirkungsgesetz fußende Analysenmethode einiger binärer Verbindungen. Zur Prioritätsfrage. Berichte der Dtsch Chem Gesellschaft.

[2] Job P (1928). Formation and stability of inorganic complexes in solution. Ann Chim.

[3] Vosburgh WC, Cooper GR (1941). Complex Ions. I. The identification of complex ions in solution by spectrophotometric measurements. J Am Chem Soc.

[4] Qin Z, Niu W, Tan R (2009). Spectrophotometric method for the determination of telmisartan with congo red. J Anal Chem.

[5] Zayed SIM (2009). Two charge-transfer complex spectrophotometric methods for the determination of sulpiride in pharmaceutical formulations. Cent Eur J Chem.

[6] Shaalan RA-A (2010). Improved spectrofluorimetric methods for determination of penicillamine in capsules. Cent Eur J Chem.

[7] Nagaraj P, Aradhana N, Shivakumar A (2009). Spectrophotometric method for the determination of chromium (VI) in water samples. Environ Monit Assess.

[8] Ostović D, Stelmach C, Hulshizer B (1993). Formation of a chromophoric complex between alendronate and copper(II) ions. Pharm Res.

[9] Kuljanin J, Janković I, Nedeljković J (2002). Spectrophotometric determination of alendronate in pharmaceutical formulations via complex formation with Fe(III) ions. J Pharm Biomed Anal.

[10] Likussar W, Boltz DF (1971). Theory of continuous variations plots and a new method for spectrophotometric determination of extraction and formation constants. Anal Chem.

[11] Yoe JH, Jones AL (1944). Colorimetric determination of iron with disodium-1,2-dihydroxybenzene-3,5-disulfonate. Ind Eng Chem Anal Ed.

[12] Harvey AE, Manning DL (1950). Spectrophotometric methods of establishing empirical formulas of colored complexes in solution. J Am Chem Soc.

[13] Diehl H, Lindstrom F (1959). Eriochrome black t and its calcium and magnesium derivatives. Anal Chem.

[14] Chriswell CD, Schilt AA (1975). New and improved techniques for applying the mole ratio method to the identification of weak complexes in solution. Anal Chem.

[15] Nan Z, Chun-Xiang H (1993). Improved mole ratio method by dual-wavelength spectrophotometry. Analyst.

[16] Mansour F, Danielson N (2012). Ligand exchange spectrophotometric method for the determination of mole ratio in metal complexes. Microchem J.

[17] Mabrouk M, Hammad SF, Abdelaziz MA, Mansour FR (2018). Determination of etidronate in pharmaceutical formulations by RP-HPLC method with indirect UV detection. Arab J Med Sci.

[18] Mansour FR, Shafi MA, Danielson ND (2012). Flow injection determination of carboxylate, phosphate, and sulfhydryl compounds using metal exchange complexation. Talanta.

