# Supplementary figures and images for: Ligand exchange method for determination of mole ratios of relatively weak metal complexes: a comparative study
Source: Chem Cent J. 2018 Dec 20;12:143. doi: 10.1186/s13065-018-0512-4 (PMC6768135; doi:10.1186/s13065-018-0512-4)

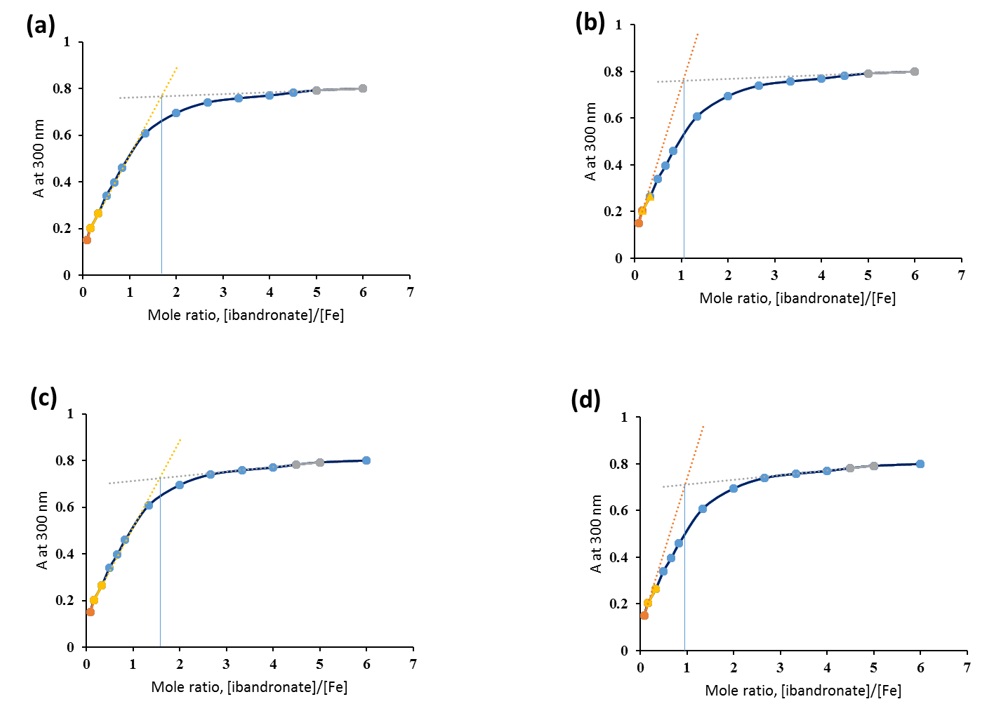

Supplement: Supplementary file 2 — Additional file 2: Fig. S1. Molar ratio’s plots for Fe(III) complex with ibandronate showing different conclusions for the same results depending on the drawn tangents. [file 13065_2018_512_MOESM2_ESM.jpg]
